# Identification of novel key genes and potential candidate small molecule drugs in diabetic kidney disease using comprehensive bioinformatics analysis

**DOI:** 10.3389/fgene.2022.934555

**Published:** 2022-08-12

**Authors:** Bin Li, Siyang Ye, Yuting Fan, Yi Lin, Suchun Li, Huajing Peng, Hui Diao, Wei Chen

**Affiliations:** ^1^ Department of Nephrology, The First Affiliated Hospital, Sun Yat-sen University, Guangzhou, China; ^2^ NHC Key Laboratory of Clinical Nephrology (Sun Yat-sen University) and Guangdong Provincial Key Laboratory of Nephrology, Guangzhou, China

**Keywords:** diabetic kidney disease, bioinformatics analysis, small-molecule drugs, differentially expressed genes, novel biomarkers, yes-associated protein 1

## Abstract

**Objective:** The currently established diagnostic and prognostic tools for diabetic kidney disease (DKD) have limitations, which demands the necessity to find new genes and pathways associated with diagnosis and treatment. Our study aims to reveal the gene expression alteration and discover critical genes involved in the development of DKD, thus providing novel diagnostic molecular markers and therapeutic targets.

**Materials and methods:** The differences of infiltrating immune cells within kidney were compared between healthy living donors and DKD patients. Besides, differentially expressed genes (DEGs) within kidney from healthy living donor, early stage DKD and advanced stage DKD samples were detected. Furthermore, the weighted co-expressed network (WGCNA) and protein-protein interaction (PPI) network were constructed, followed by recognition of core hub genes and module analysis. Receiver operating characteristic (ROC) curve analysis was implemented to determine the diagnostic value of hub genes, correlation analysis was employed to explore the association between hub genes and infiltrating immune cells, and certain hub genes was validated by quantitative real-time PCR and immunohistochemistry staining in cultured tubule cells and diabetic mice kidney. Finally, the candidate small molecules as potential drugs to treat DKD were anticipated through utilizing virtual screening and molecular docking investigation.

**Results:** Our study revealed significantly higher proportion of infiltrating immune cells within kidney from DKD patients via probing the immune landscape by single-cell transcriptomics. Besides, 126 commonly shared DEGs identified among three group samples were enriched in immune biological process. In addition, the ROC curve analysis demonstrated the strong diagnostic accuracy of recognized hub genes (*NFKB1*, *DYRK2*, *ATAD2*, *YAP1*, and *CHD3*) from PPI network. Correlation analysis further confirmed the positive association between these hub genes with infiltrating natural killer cells. More importantly, the mRNA transcripts and protein abundance of YAP1 were significantly higher in high glucose-treated renal tubule cells and diabetic mice kidney, and the small molecules exhibiting the best binding affinities with YAP1 were predicted and acquired.

**Conclusion:** Our findings for the first time indicate that *NFKB1*, *DYRK2*, *ATAD2*, *YAP1*, and *CHD3* might be potential novel biomarkers and therapeutic targets for DKD, providing insights into the molecular mechanisms underlying the pathogenesis of DKD.

## Introduction

Diabetes mellitus (DM) has emerged as a global epidemic and the worldwide prevalence of adult DM has reached 415 million (Ogurtsova et al., 2017). Diabetes kidney disease (DKD) is among the most severe complications related to DM, affecting 25 percent of type 1 DM and 40 percent of type 2 DM, respectively (Callaghan et al., 2012). DKD is characterized by increased urine albumin excretion and microalbuminuria, as well as diminished renal function, as shown by the increased plasma creatinine concentration or diminished glomerular filtration rate (Fineberg et al., 2013). DKD leads to a significant percentage of end-stage renal disease (ESRD) and eventually results in renal replacement therapy in developed countries (Yung et al., 2013; Alicic et al., 2017). Despite the current use of angiotensin converting enzyme inhibitors and angiotensin II receptor blockers, the risk of DKD progression has still not been lowered (Bash et al., 2008; Dounousi et al., 2015), pointing to huge unsatisfied demand for innovative therapies for DKD. Of note, a clinically silent early stage DKD develops along the course of disease before the manifestation of advanced stage DKD (Bjornstad et al., 2014; Chen L. et al., 2020), thus there is an unmet need for discovering better diagnostic molecular markers that can early identify individuals at high risk of DKD progression, as well as better therapeutic targets for optimal treatment to prevent the development to advanced stage DKD.

DKD is one of the most serious microvascular complications of DM that is linked with systemic or renal inflammation (Winter et al., 2018). Classic inflammatory biomarkers and effector molecules, as well as immune cells, are dramatically increased within the renal microenvironment of DKD patients (Mensah-Brown et al., 2005). For instance, infiltrating T and B cells were shown to be much higher in type 2 diabetic human kidneys and were positively associated with the degree of proteinuria (Moon et al., 2012). The infiltration and activation of immune cells within kidney microenvironment contributes to the acceleration of chronic inflammation, renal damage, and advancement of DKD (Matoba et al., 2019). Accordingly, dysregulated renal immune status and overwhelming inflammation possibly serve as indicators for early detection and monitoring of disease progression, as well as potential targets for therapeutic intervention. On the other side, the integrated use of bioinformatics approaches based on high-throughput techniques has enabled the investigation of significantly altered genes that are closely linked with immune cell infiltration and activation (Li et al., 2020; Li et al., 2022). However, to the best of our knowledge, there are few studies that employed bioinformatic tools to analyze the existing multiple genomics data and find new potential biomarkers or therapeutic targets associated with immune status alteration within DKD kidney.

**FIGURE 1:**
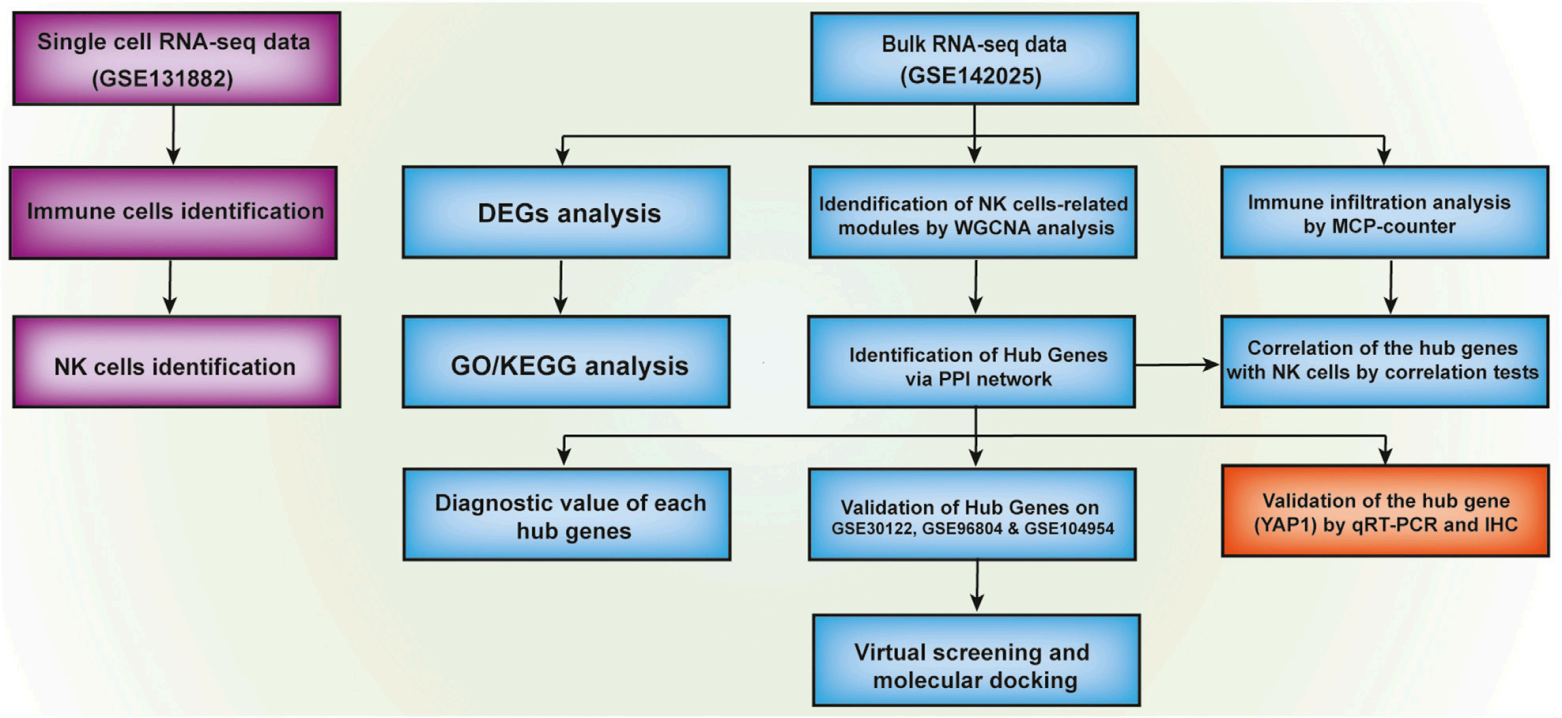
Flowchart of the analysis used in this study.

The objective of our current research is to comprehensively analyze the transcriptomic profiles of the existing DKD-related datasets for a deeper understanding of the pathogenesis of DKD (Figure 1). Findings from our investigation indicated a significantly higher proportion of immune cells including natural killer (NK) cells in diabetic kidney in comparison with healthy living donor by using Single-cell sequencing (scRNA-seq) dataset GSE131882. Besides, using public transcription profiling by array data (GSE142025) including kidney samples from DKD patients and healthy living donor, we for the first time identified five novel hub genes (*NFKB1*, *DYRK2*, *ATAD2*, *YAP1*, and *CHD3*) that were significantly linked with the immune status such as NK cells infiltration and validated by Receiver operating characteristic (ROC) curve analysis. More importantly, significantly higher YAP1 transcripts and protein expression were validated in high glucose-treated tubule cells and diabetic mouse kidney tissue. Finally, two small molecules with the highest binding affinities to YAP1 were screened by molecular docking.

## Materials and methods

### Retrieval of single-cell RNA sequencing dataset collection

The scRNA-seq data was derived from the Gene Expression Omnibus (GEO) dataset with the accession number GSE131882 that includes 23,980 single-cell transcriptomes from three healthy kidney samples and three diabetic kidney samples, respectively. The analysis of scRNA-seq data was conducted by the R package Seurat (Song et al., 2022) and data filtering and normalization were carried out. Cells with a unique feature count of more than 2,000, fewer than 200 or with a mitochondrial count of more than 10% were eliminated (Supplementary Figure S1), and the remaining 20,552 cells were used for subsequent analysis. Moreover, unsupervised clustering was performed *via* uniform manifold approximation and projection (UMAP) and t-distributed stochastic neighbor embedding (t-SNE), while the immune cell cluster was manually annotated using the known immune cell marker genes (*CD3G*, *CD8A*, *FCGR3A*, and *NCAM1*). Then, immune cells were selected for the subcluster analysis. Among the identified subclusters, we annotated the NK cell cluster by known NK cell marker genes (*KLRD1, IL2RB, FCGR3A,* and *SLAMF6*).

### Retrieval of microarray dataset collection, identification of differentially expressed genes and gene ontology analyses

The high throughput gene expression profile of GSE142025 dataset that contains kidney biopsy from 9 healthy living donor, 6 early stage DKD and 22 advanced stage DKD samples were obtained from GEO and firstly standardized, normalized by Transcripts Per Kilobase Million (Fan et al., 2019). The R edgeR package was used to identify differentially expressed genes (DEGs) (Robinson et al., 2010). Genes with log2foldchange > 0.8 and *p*-value < 0.05 were defined as DEGs between healthy living donor and early stage DKD or advanced stage DKD samples, while genes with log2foldchange > 1.5 and *p*-value < 0.05 were defined as DEGs between early stage DKD and advanced stage DKD samples. Volcano plots and heatmap clusters of the DEGs were plotted using the ggplot modules of R package. The overlapping DEGs among all three group samples were further screened to define the commonly shared DEGs essential for the pathogenesis of DKD, and these commonly shared DEGs were subjected to Gene Ontology (GO) and Kyoto Encyclopedia of Genes and Genomes (KEGG) pathway enrichment analysis through clusterprofiler package.

### Evaluation of immune cells infiltration

Microenvironment Cell Populations-counter (MCP-counter) R package, a transcriptome-based computational method that robustly quantifies the absolute infiltration abundance of eight immune (CD3^+^ T cells, CD8^+^ T cells, cytotoxic lymphocytes, NK cells, B lymphocytes, cells originating from monocytes (monocytic lineage), myeloid dendritic cells, neutrophils), as well as two stromal cell subpopulations (endothelial cells and fibroblasts) in a heterogeneous tissue sample was employed in our current study (Becht et al., 2016). From a gene expression matrix, MCP-counter calculates an abundance score for each cell subtype in each sample, and these scores can be utilized for comparing the abundance of each cell subpopulations across samples within a cohort. In our current study, the T cells, NK cells and fibroblasts were selected for further analysis.

### Weighted co-expression network and protein-protein interaction network

A weighted gene co-expression network (WGCNA) was implemented *via* the WGCNA package in R language for transcriptomic data of GSE142025 (Langfelder and Horvath, 2008). A similarity matrix was constructed by calculating the correlation coefficient between any two genes, and the similarity matrix was subsequently converted into an adjacency matrix according to the optimal soft threshold. The cutreeDynamic function was used for tree pruning of the gene hierarchical clustering dendrograms resulting in co-expression modules. Modules with a similarity >0.75 were then combined into a single module with a minimum of 50 genes. Association between eigenvalues and immune status was assessed by Pearson’s correlation, and the modules with the strongest associations to immune cells was selected as candidate module for additional investigation. The STRING database (http://stringdb.org/) was applied to establish the module genes-encoded proteins and their connections. Cytoscape was used to design and test the protein-protein interaction (PPI) network, and the degree was utilized to rank and select hub genes by *cytoHubba* plugin. The diagnostic accuracy values of selected hub genes were determined by ROC curve analysis from the pROC package (Robin et al., 2011).

### Correlation analysis between differentially expressed genes and infiltrating immune cells

The commonly shared DEGs among three groups were regarded as critical regulators linked with the development of DKD. Accordingly, the ggstatsplot package was utilized to implement correlation analysis between these commonly shared DEGs and infiltrating immune cells.

### Validation of hub genes on independent datasets

The high throughput gene expression profiles of GSE30122 (Na et al., 2015), GSE96804 (Pan et al., 2018), and GSE104954 (Grayson et al., 2018) datasets that contain kidney biopsy from healthy living donor and DKD samples were obtained and used for validation of hub genes. GSE30122 contains 10 DKD glomeruli and 24 control glomeruli samples, GSE96804 contains 41 DKD kidney and 20 control samples, and GSE30122 contains 7 DKD kidney and 18 control samples.

### Cell culture

Human proximal tubular cell line, human kidney 2 (HK-2) cells purchased from American Type Culture Collection (ATCC, Manassas, VA, United States ) were cultured in Dulbecco’s Modified Eagle Medium/Nutrient Mixture F-12 supplemented with 10% fetal bovine serum (FBS), 1% penicillin/streptomycin at 37°C in 5% Co_2_. Cells were growth arrested in culture media containing 0.5% FBS for 12 hours before the initiation of all experiments. For high glucose (HG) administration, HK-2 cells were subjected to 5.5 mM glucose as normal treatment or 45 mM glucose as HG treatment.

### RNA isolation and quantitative real-time PCR

Trizol reagent (Life Technologies Corporation, Carlsbad, CA, United States) was utilized to extract the total RNA from HK-2 cells. Isolated RNA was utilized for synthesizing cDNA by High-Capacity cDNA Reverse Transcription Kit (Applied Biosystems, Carlsbad, CA, United States). Quantitative real-time PCR (qRT-PCR) analyses were performed in the StepOnePlus Real-time PCR Systems (Applied Biosystems) using SYBR Green Master Mix (Applied Biosystems). The following sets of primers were used: *β-ACTIN*: 5′-GCA​CAG​AGC​CTC​GCC​TT-3′ (forward) and 5′-GTT​GTC​GAC​GAC​GAG​CG-3′ (reverse); *YAP1*: 5′-TGA​CCC​TCG​TTT​TGC​CAT​GA-3′ (forward) and 5′-GTT​GCT​GCT​GGT​TGG​AGT​TG-3′ (reverse). Template cDNA was added to each PCR reaction and each biological sample was conducted in technical duplicates for each gene. The thermal cycling conditions consisted of pre-denaturation at 95°C for 2 min; 40 cycles of 95°C for 30 s, 58°C for 30 s, and 72°C for 60 s; and a final extension at 72°C for 5 min. Relative quantification of genes in each individual sample was normalized to *β-ACTIN* expression using StepOne software v2.3 (Applied Biosystems).

### Animal experiment

Male mice (6 weeks old, 20 ± 2 g) were purchased from Shanghai Model Organisms Center (Shanghai, China). All mice were kept under standard conditions (23°C ± 2°C, 60% humidity) with a 12 h light-dark cycle and allowed free access to food and water. All animal procedures were approved by the Animal Care and Use Committee and performed in accordance with the ethical guidelines of Sun Yat-sen University (Approval Number: 2021001252). Diabetic kidney mice were induced by high-fat diet (Research Diets, New Brunswick, NJ, United States) for 8 weeks followed by a single intraperitoneal injection of streptozotocin at a dosage of 35 mg/kg/day for five consecutive days, whereas the control mice were received common normal diet (Research Diets) and injected intraperitoneally with equal volume of citrate buffer. After the completion of the high-fat diet for another 8 weeks, mice were deeply anesthetized (200 mg/kg ketamine hydrochloride, 10 mg/kg xylazine, 0.2 mg/kg acetylpromazine) and euthanized by decapitation. Kidneys were excised, snap-frozen in liquid nitrogen, and stored at −80°C until use.

### Immunohistochemistry staining

Paraffin-embedded kidney section was deparaffinized and rehydrated, followed by microwave-based antigen retrieval in citrate buffer. Sections were quenched by 3% hydrogen peroxide and blocked with 2% bovine serum albumin. The primary antibody against YAP1 (ab205270, Abcam, Cambridge, United Kingdom) was added according to the instructions and incubated at 4°C for 12 h. The secondary antibody (ab205718, Abcam) was added and incubated at room temperature for 10 min. DAB (Dako, Carpinteria, CA, United States) was added and counterstained for 5 min, and the staining was observed under a microscope. Quantification of staining was performed using ImageJ analysis software.

### Virtual screening and molecular docking protocol

The protein structure of YAP1 protein was determined by AlphaFold2 that is capable of learning far more effectively with little data and producing correct structure models without known templates (Jumper et al., 2021). The structure of 1615 FDA approved small molecule drugs were downloaded from zicn15 database (Sterling and Irwin, 2015). The virtual screening was applied on the AutoDock Vina that is an application of PyRx (Dallakyan and Olson, 2015). The parameters in virtual screening were set as “center_x = −3.53547519904, center_y = 11.7871326158, center_z = 10.6340273361, size_x = 62.2605676947, size_y = 43.6242652316, and size_z = 40.4080936428”. Two small molecules with the lowest binding free energy (low binding free energy represents a stable protein-molecule complex) were selected as two best docking models with the largest ligand-binding affinities. The molecular docking was completed on Autodock4 software, and the docking results were plotted by Pymol software.

## Results

### Immune cells increased in kidney tissue from Diabetes kidney disease samples

**FIGURE 2:**
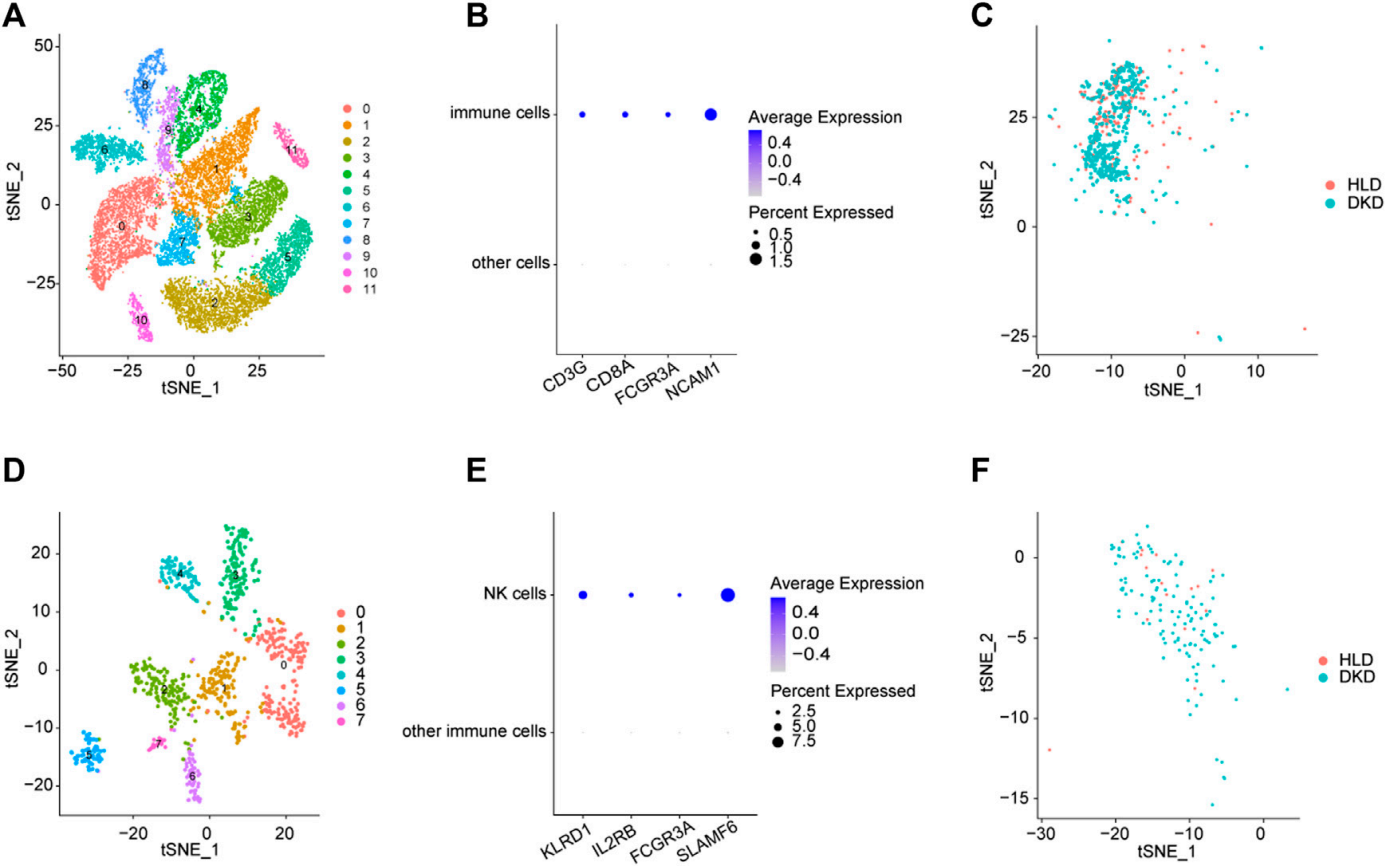
Integrated scRNA-seq of kidneys from healthy living donors and diabetic samples. **(A)** The identified clusters by TSNE analysis. **(B)** The expression patterns of immune cells biomarkers. **(C)** The distributions of immune cell clusters. **(D)** The identified subclusters among immune cells by TSNE analysis. **(E)** The expression patterns of NK cells biomarkers. **(F)** The distributions of NK cell clusters. HLD, healthy living donor; DKD, diabetic kidney disease.

An unsupervised clustering technique was employed to recognize 12 distinct cell types by using the scRNA-seq dataset of GSE131882. Among these cell clusters, cluster 9 had a greater number of immune cell markers and was therefore designated as an immune cell cluster (Figures 2A,B). Cluster 9 was comprised of 860 cells, 602 of which were from DKD samples and 258 of which were from healthy living donor samples (Figure 2C), indicating a significantly elevated infiltrating immune cells within kidney microenvironment of DKD patients. By further subcluster analysis on the immune cell cluster, we found that there were eight subclusters among immune cells, and subcluster 2 had a greater number of NK cell markers and was therefore designated as the NK cells (Figures 2D,E). Subcluster 2 was comprised of 144 NK cells, 129 of which were from DKD samples and 15 of which were from healthy living donor samples (Figure 2F), indicating a significantly elevated infiltrating NK cells within kidney microenvironment of DKD patients. Consistently, by using the MCP-counter technique to quantify the absolute abundance of immune cells, we also found robustly increased values of NK cells within kidney from both early stage DKD and advanced stage DKD samples (Supplementary Figure S2).

### Identification of differentially expressed genes

**FIGURE 3:**
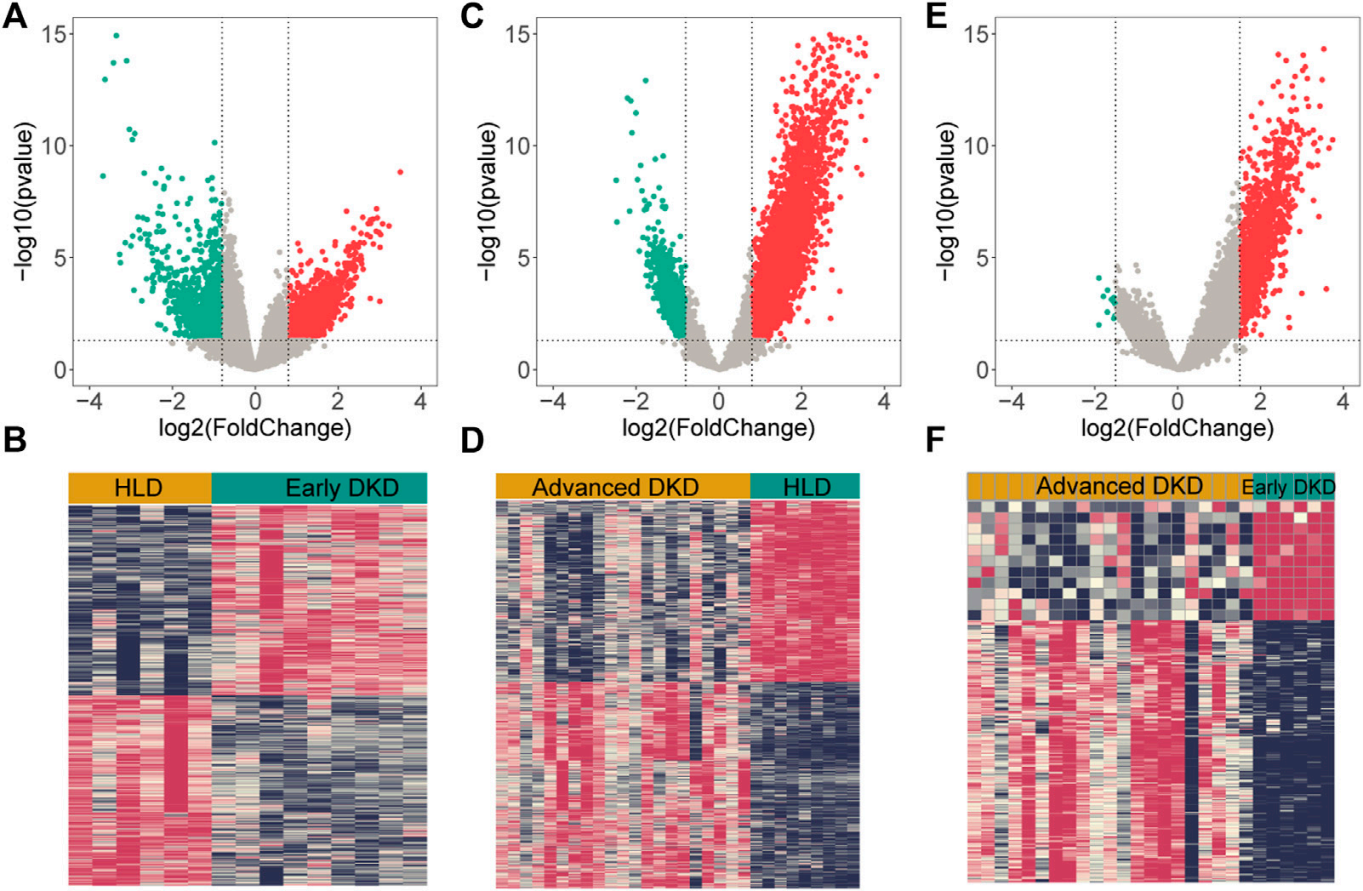
The volcano and heatmap plots of differentially expressed genes (DEGs). Red points indicate up-regulated genes, green points indicate down-regulated genes, and gray points indicate genes with no significant difference. **(A,B)** DEGs from 9 healthy living donors and 6 early stage DKD samples. **(C,D)** DEGs from 9 healthy living donors and 21 advanced stage DKD samples. **(E,F)** DEGs from 6 early stage DKD and 21 advanced stage DKD samples. HLD, healthy living donor; DKD, diabetic kidney disease.

**FIGURE 4:**
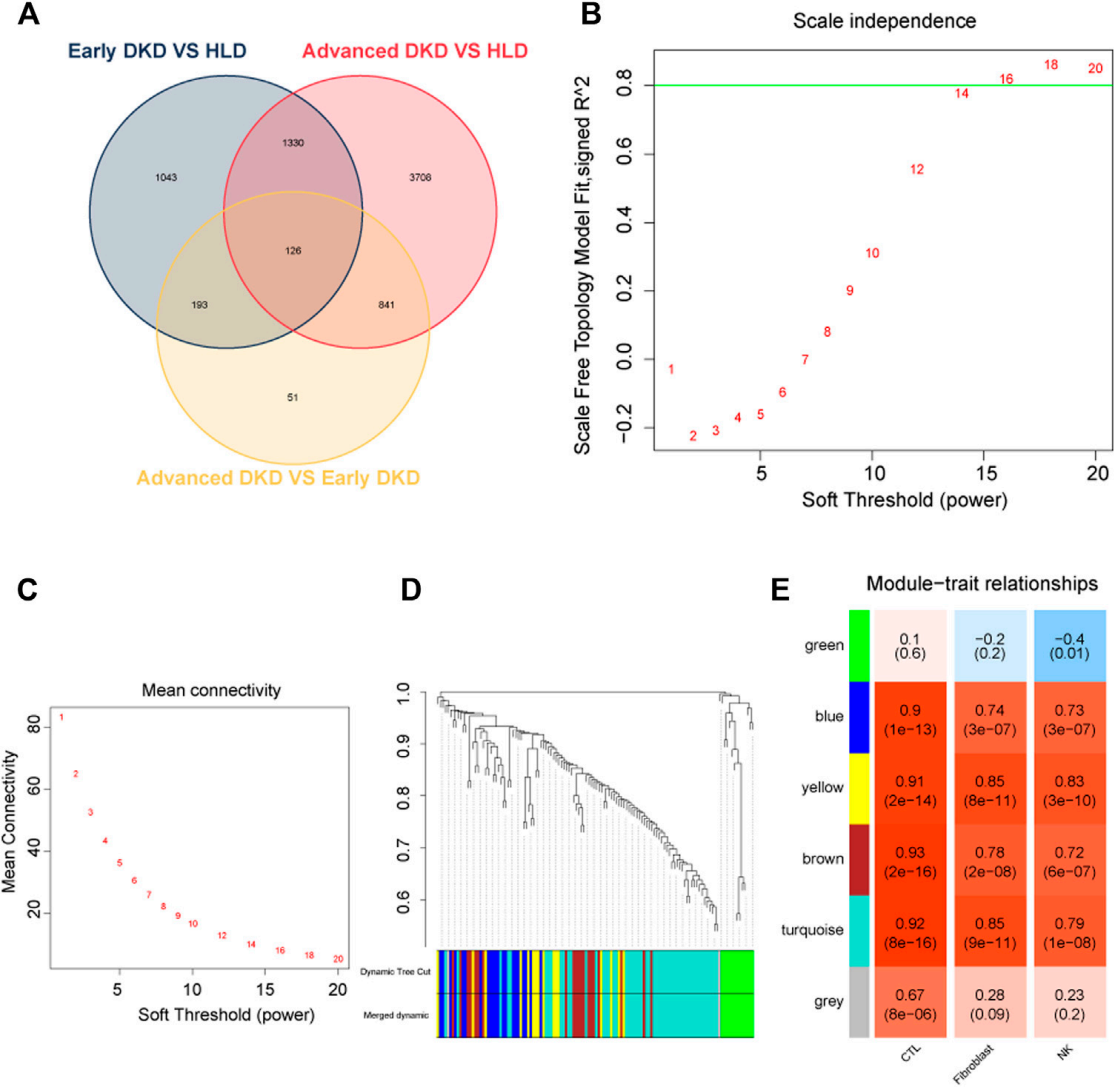
Weighted co-expressed network (WGCNA) analysis. **(A)** Venn diagram for differentially expressed genes. **(B)** A graph showing the selection of a soft threshold. **(C)** Under different soft thresholds, the mean connectivity of genes is calculated. **(D)** The original module (dynamic tree cut) and allocated merged modules (merged dynamic). **(E)** Module-trait links. Each row corresponds to a module eigengene, and the column corresponds to cytotoxic T lymphocytes (CTLs), fibroblast, natural killer (NK) cells. Correlation and *p*-value were displayed for each cell subtype. HLD, healthy living donor; DKD, diabetic kidney disease.

Upon setting the cut-off criterion as *p*-values < 0.05 and log2foldchange > 0.8, 2,692 DEGs (1,518 upregulated and 1,174 downregulated) between the 9 healthy living donors and 6 early stage DKD samples were recognized in GSE142025 dataset, and the volcano and heatmap plots of DEGs were plotted in Figures 3A,B. Meanwhile, 6,005 DEGs (4,033 upregulated and 1,972 downregulated) between the 9 healthy living donors and 21 advanced stage DKD samples were recognized based on *p*-values < 0.05 and log2foldchange > 0.8, with the volcano and heatmap plots of DEGs displayed in Figures 3C,D. Moreover, 1,211 DEGs (1,200 upregulated and 11 downregulated) between the 6 early stage DKD samples and 21 advanced stage DKD samples were found based on *p*-values < 0.05 and log2foldchange >1.5, with the volcano pot and heatmap plot DEGs presented in Figures 3E,F. In addition, the Venn diagram was implemented to exhibit the commonly shared 126 DEGs among samples from three different disease stages (Figure 4A).

### Gene ontology and pathway enrichment analysis of commonly shared differentially expressed genes

The commonly shared DEGs among three group samples in GSE142025 dataset were uploaded to clusterprofiler package to perform GO and KEGG pathway enrichment analysis. The most enriched GO terms in biological process (BP) term included various immune processes such as neutrophil activation, immune response-activating signal transduction, T cell activation, and mononuclear cell differentiation (Supplementary Table S1), which to some extent agreed with our previous findings showing the significantly elevated infiltrating immune cells within kidney microenvironment of DKD patients (Figure 2). The primarily enriched KEGG pathways contained B cell receptor signaling, tumor necrosis factor (TNF) signaling, the cell adhesions, and T cell receptor signaling (Supplementary Table S2).

### Construction of the weighted co-expressed network

In WGCNA analysis, the expression data of commonly shared DEGs was utilized to find the co-expressed gene modules. A soft threshold (*β*) = 16 (Figures 4B,C) was selected to ensure a scale-free network (*R2* = 0.84; Supplementary Figure S3). Modules having a height cut-off value of 0.25 were deemed similar and chosen for further integration (Figure 4D). Six modules (colored by green, blue, yellow, brown, turquoise, and gray) were recognized, and associations between modules and traits were determined. Interestingly, the yellow module possessed the most positive correlation with NK cells (*r* = 0.83) and fibroblasts (*r* = 0.85) with the lowest *p*-values (Figure 4E).

### Identification and evaluation of hub genes

**FIGURE 5:**
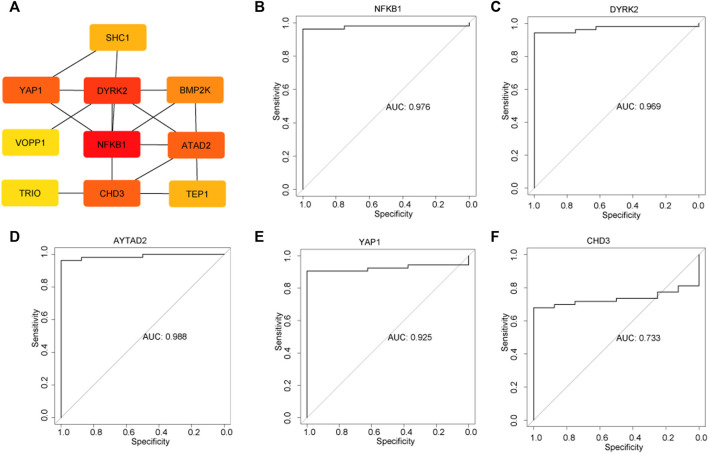
Identification of hub genes and the evaluation the diagnostic value of each hub gene. **(A)** Top five hub genes were selected by the Cytohub program. The AUC values of five hub genes: *NFKB1* **(B)**, *DYRK2* **(C)**, *AYTAD2* **(D)**, *YAP1* **(E)**, *CHD3* **(F)**.

Subsequently, the 16 genes from the yellow module were uploaded into the STRING platform to construct a PPI network of 13 nodes and 26 edges (Figure 5A). Five genes with a higher degree value, including nuclear factor kappa B subunit 1 (*NFKB1*), dual specificity tyrosine phosphorylation regulation kinase 2 (*DYRK2*), ATPase family AAA domain containing 2 (*ATAD2*), yes-associated protein 1 (*YAP1*) and chromodomain helicase DNA binding protein 3 (*CHD3*), were recognized as hub genes that play key roles in the pathogenesis of DKD. ROC curves were employed to evaluate the diagnostic value of each hub gene, with the AUC values being 0.976, 0.969, 0.988, 0.925, and 0.733 for *NFKB1*, *DYRK2*, *ATAD2*, *YAP1,* and *CHD3*, respectively (Figures 5B–F), confirming the capacity of these hub genes to distinguish DKD from healthy living donors.

### Expression patterns of hub genes

**FIGURE 6:**
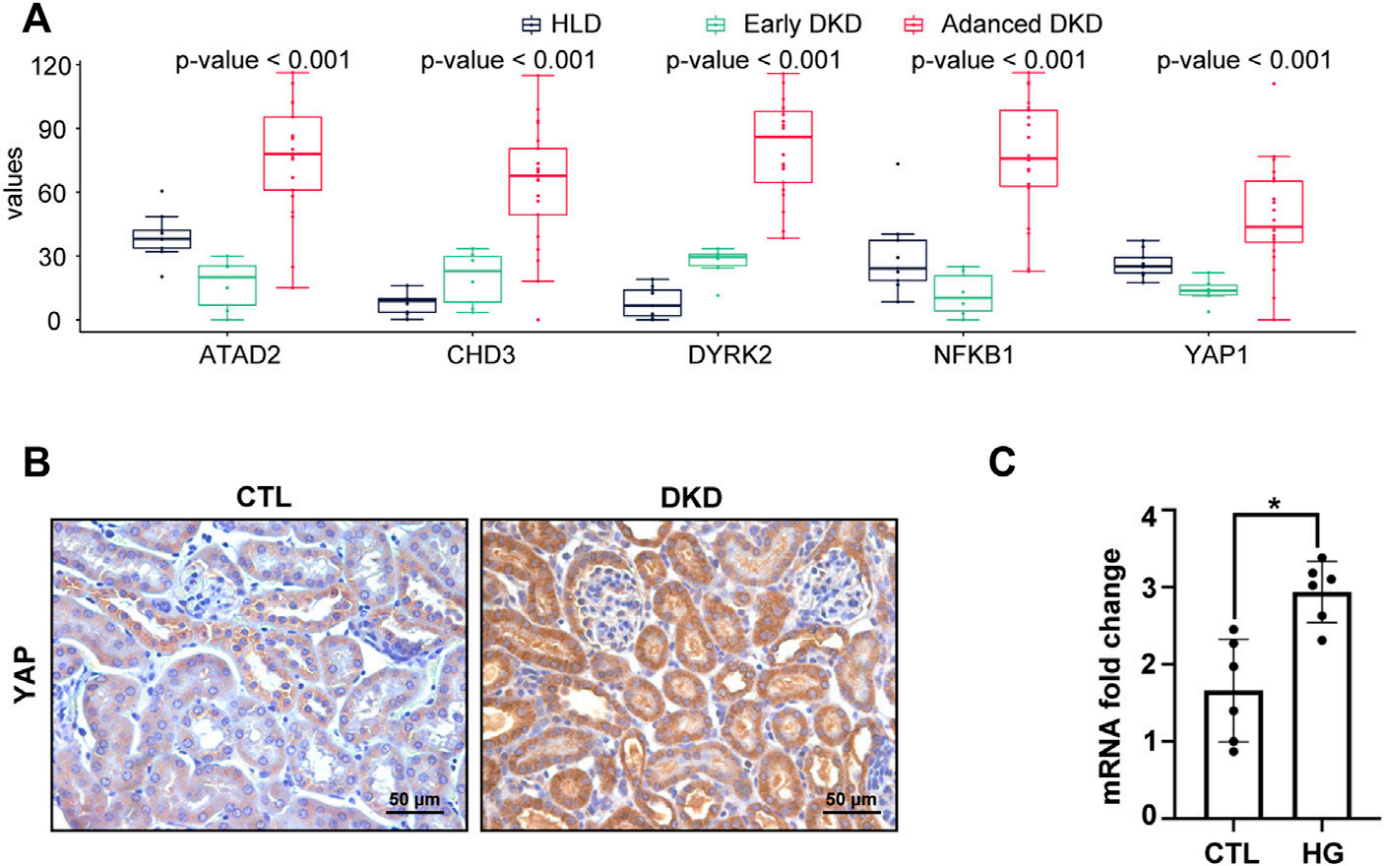
YAP1 was increased in advanced stage DKD samples, high glucose treated HK-2 cells, and mice kidney of diabetic kidney disease. **(A)** The expression patterns of hub genes among healthy living donor, early stage DKD, and advanced stage DKD samples. **(B)** Representative IHC staining of YAP1 on mice kidney section of diabetic kidney disease. **(C)** High glucose treatment increased the mRNA expression in HK-2 cells. For all graphs, results were expressed as mean ± SD of data from three experiments. *p < 0.05 versus control (CTL). CTL, control; HG, high glucose; HLD, healthy living donor; DKD, diabetic kidney disease.

Analysis of all the five hub genes (*NFKB1*, *DYRK2*, *ATAD2*, *YAP1,* and *CHD3*) from GSE142025 dataset revealed that all of them reached the highest expression levels in the kidney tissues of advanced stage DKD samples (Figure 6A). Besides, the expression levels of *CHD3* and *DYRK2* were minimized in the kidney tissues of healthy living donor samples (Figure 6A). By contrast, the kidney tissues of early stage DKD samples had the lowest expression levels of *ATAD2*, *NFKB1*, and *YAP1* (Figure 6A).

### Validation of hub genes on another three independent datasets

Then, the five recognized hub genes were further validated in another three bulk transcriptomic datasets from kidney tissue. In accordance with our previous findings from GSE142025, *CHD3, DYRK2,* and *NFKB1* were significantly higher in DKD samples from GSE30122 (Supplementary Figure S4), *NFKB1* and *ATAD2* were found dramatically elevated in DKD samples from GSE96804 (Supplementary Figure S5), and *ATAD2, DYRK2,* and *NFKB1* substantially increased in DKD samples from GSE104954 (Supplementary Figure S6).

### Validation of yes-associated protein 1 expression, and correlation of the hub genes with natural killer cell

**FIGURE 7:**
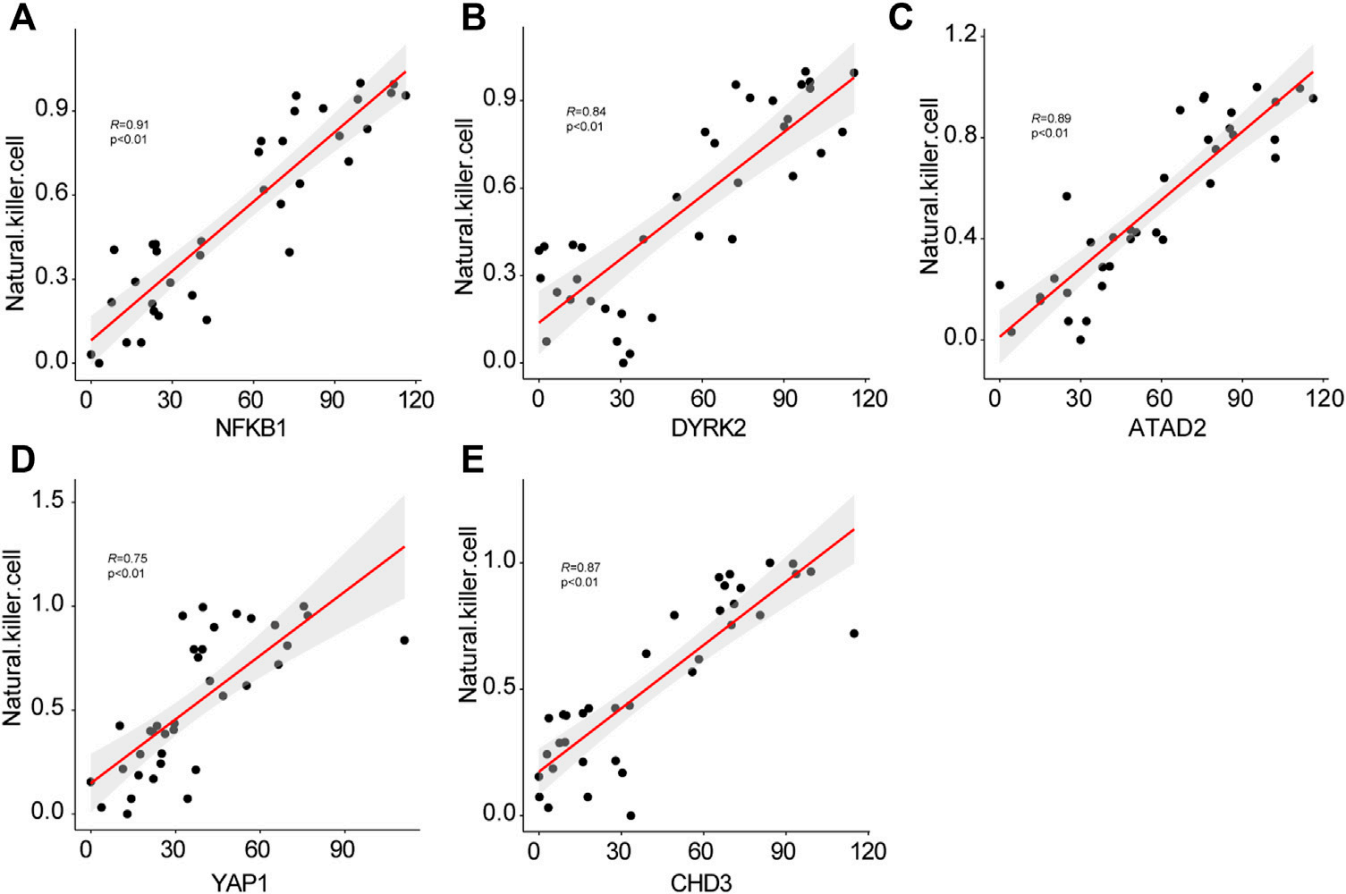
The correlations of hub genes *NFKB1* **(A)**, *DYRK2* **(B)**, *AYTAD2* **(C)**, *YAP1* **(D)**, *CHD3* **(E)** with infiltrating natural killer cells.

To verify the constant shift in *YAP1* expression in DKD, we evaluated the mRNA and protein expression levels of *YAP1* using qRT-PCR and immunohistochemistry staining. Consistent with the highest expression of *YAP1* gene in the kidney tissues of advanced stage DKD samples (Figure 6A), *YAP1* transcripts were found to be significantly elevated in cultured tubular cells treated with high glucose (Figure 6C), and a greater amount of YAP1 was detected in tubular cells from DKD mice kidney (Figure 6B). Moreover, our data revealed that the expression levels of *NFKB1*, *DYRK2*, *ATAD2*, *YAP1* and *CHD3* were positively correlated with NK cell infiltration, with the correlation values being 0.91, 0.84, 0.89, 0.75, and 0.87 for *NFKB1*, *DYRK2*, *ATAD2*, *YAP1,* and *CHD3*, respectively (Figures 7A–E). The consistence of enhanced YAP1 expression under diabetic conditions with previous bioinformatics analysis, together with the powerful associations between hub genes and NK cells, suggested the essential roles of these hub genes in triggering the dysregulated immune status. From the overall analysis of hub genes and expression analysis, overexpression of *DYRK2* and *CHD3* could be the potential diagnostic biomarkers for early stage DKD, while overexpression of *NFKB1*, *ATAD2* and *YAP1* could be the possible prognostic biomarkers for advanced stage DKD. Particularly, the obviously upregulated YAP1 might represent a potential therapeutic target for DKD.

### Virtual screening and molecular docking

**FIGURE 8:**
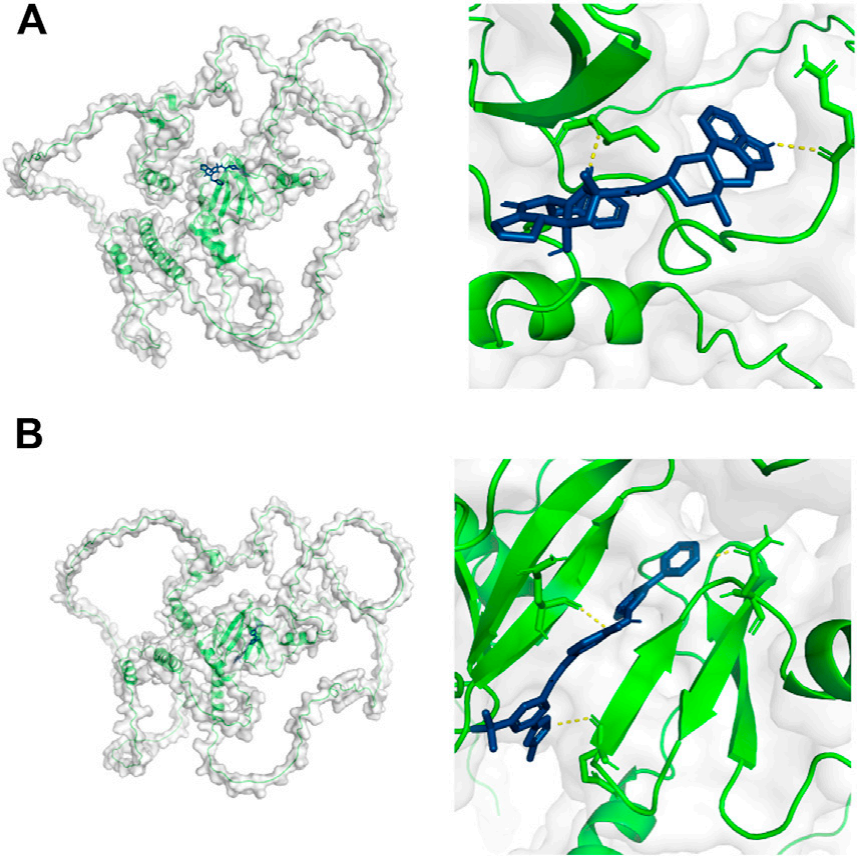
Molecular docking results of YAP1 with ZINC000003978005 **(A)** and ZINC000006716957 **(B)**. Green color represents the structure of YAP1 protein, the blue color represents the structure of small molecules, and the yellow color represents the hydrogen bonds between the YAP1 protein and small molecules.

The virtual screening results from Vina software showed that the higher binding abilities of these small molecules were ZINC000003978005 (−9.7 kcal/mol), ZINC000006716957 (−9.7 kcal/mol), ZINC000242548690 (−9.6 kcal/mol), ZINC000150338819 (−9.4 kcal/mol), ZINC000169289767 (−9.2 kcal/mol), and ZINC000164760874 (−9.0 kcal/mol). Then, ZINC000003978005 and ZINC000006716957 were submitted to the molecular docking analysis. The bindings of these two small molecules with YAP1 were shown in Figures 8A,B.

## Discussion

DKD is one serious microvascular complication of long-term DM associated with growing global public health and economic burden (Tang and Yiu, 2020). Nevertheless, current therapies such as renin-angiotensin-aldosterone system or sodium-glucose cotransporter 2 inhibitors cannot impede the malignancy of DKD, partly due to the incomplete understanding of the complicated etiology. Therefore, it is of great importance to explore the new molecular mechanisms, find novel key genes, biomarkers and potential small-molecule inhibitors that may be helpful for the diagnosis, monitoring and treatment of DKD.

Our current study aims to reanalyze the omics molecular profiles available in the public databases to recognize the potential diagnosis, monitoring and prognosis biomarkers, as well as small-molecule chemical perturbagens for DKD (Figure 1). We firstly found the apparently elevated infiltrating NK cells in kidney tissues from DKD patients using the scRNA-seq dataset. Secondly, a total of 126 commonly shared DEGs were identified using bulk RNA-seq data of kidney tissues from healthy living donor, early stage DKD and advanced stage DKD patients. Gene annotations of these commonly shared DEGs revealed upregulations of immune-related biological processes such as neutrophil activation, immune response-activating signal transduction, T cell activation, and mononuclear cell differentiation, suggesting a crucial role of immune cell-mediated proinflammatory status in the development of DKD. Subsequently, five novel hub genes (*NFKB1*, *DYRK2*, *ATAD2*, *YAP1*, and *CHD3*) were recognized and demonstrated the strong diagnostic accuracy of distinguishing DKD patients from healthy living donors. Intriguingly, correlation analysis further confirmed the positive association between these five hub genes and infiltrating NK cells. More importantly, the mRNA transcripts and protein abundance of YAP1 were in line with the bioinformatics analysis results to display the significantly higher expression level of YAP1 in high glucose-treated renal tubule cells and diabetic mice kidney.

Our study not only displayed increased proportions of immune cells in DKD kidney by unsupervised clustering technique towards the scRNA-seq dataset, but also unraveled significantly enriched immune-cell related biological processes derived from DEGs of bulk RNA-seq data, which is consistent with previous findings to imply the altered humoral and cellular immunity within DKD renal microenvironment, as well as the essential role of activated immune cells in the pathogenesis of DKD (Tang and Yiu, 2020). Specifically, NK cells are a subgroup of innate lymphocytes that are crucial for local immunological responses (Turner et al., 2019), and human NK cells in healthy kidneys serve as sentinels to help preserve the barrier integrity against infections (Bjorkstrom et al., 2016). However, NK cells also demonstrated direct cytotoxic effects on injured tubular epithelial cells in triggering and boosting the chronic inflammation by stimulating the production of proinflammatory molecules and activating other immune cells (Uchida et al., 2019). In line with the previous evidence, WGCNA analysis employed in our current investigation found that a gene module (colored by yellow) was significantly linked with NK cell infiltration, and five upregulated hub genes (*NFKB1*, *DYRK2*, *ATAD2*, *YAP1*, and *CHD3*) were further chosen as the core hub genes within this module. In addition, the correlation analysis further confirmed the obviously positive association of these five upregulated hub genes with infiltrating NK cells, supporting our hypothesis that these five novel hub genes advanced the development of DKD possibly *via* activation of immune cells and initiation of proinflammatory status within kidney microenvironment.

To date, the established noninvasive diagnostic and prognostic tools for DKD have limitations. For example, two commonly used indicators, urinary albumin-creatinine ratio and estimated glomerular filtration rate, are not sensitive enough as biomarkers to indicate the degree of renal dysfunction and injury for differentiating early stage DKD (Bjornstad et al., 2015; Said and Nasr, 2016). Furthermore, the current therapeutic options, including blood pressure optimization, optimal control of hyperglycemia and lipid levels, and maximizing the renin-angiotensin-aldosterone system blockade can only slow but not block the progression of DKD (Nathan et al., 2013). Therefore, early and specific diagnosis and innovative strategies are urgently needed to both prevent and treat DKD, and many DKD-related new biomarkers including proteins, metabolite products and genes have been discovered in the past decades. Nevertheless, most of these markers were limited by the lack of specificity and sensitivity. The development of high-throughput technologies, such as single-cell or bulk-tissue transcriptomes, is emerging as one of the most promising approaches in discovering sensitive biomarkers and specific therapeutic targets (Herzallah and Karny, 2011). Accordingly, we further verified the hub genes (*NFKB1*, *DYRK2*, *ATAD2*, *YAP1* and *CHD3*) in GSE142025 dataset, and discovered the highest expression of all the five hub genes in advanced stage DKD samples. By comparison, the minimal expression of *CHD3* and *DYRK2* were observed in healthy living donor samples, and early stage DKD samples encountered the weakest expression of *ATAD2*, *NFKB1*, and *YAP1*. These expression patterns of hub genes proposed *CHD3* and *DYRK2* as the putative early diagnostic biomarkers for DKD, while *ATAD2*, *NFKB1*, and *YAP1* as putative biomarkers for monitoring DKD progression. More importantly, the validations of these upregulated hub genes in another three transcriptomic datasets (GSE30122, GSE96804, GSE104954) strengthened their potential as diagnostic or putative biomarkers for DKD.

Particularly, our current findings agree with previous evidence to show the close association of *NFKB1* and *YAP1* with the pathogenesis of DKD. Elevated *NFKB1* mRNA expression was attributable to the development of pro-inflammatory status in children and adolescents with type 1 diabetes, which might eventually result in deterioration in renal function and, ultimately, DKD (de Melo et al., 2022). Administration of NF-κB inhibitor reduced glomerular inflammation and oxidative stress in DKD animal model (Foresto-Neto et al., 2020). *YAP1* is the Hippo pathway’s primary transcriptional coactivator and has been linked to chronic inflammation in multiple organs and tissues (Zheng et al., 2021). In accordance with our current results showing the robust elevation of YAP1 in both high glucose-treated tubule cells and diabetic mice kidney, enhanced expression and activation of *YAP1* were previously observed in renal proximal tubular epithelial cells in diabetic mice model and patients (Chen J. et al., 2020), while inducible *YAP1* deletion or pharmacologically inhibition of YAP1 dramatically reduced tubulointerstitial inflammation and fibrosis in DKD mice (Zheng et al., 2021). Hence, findings from our study precipitates one hypothesis that enhanced YAP1 in renal tubular epithelial cells might produce inflammatory and adherence factors to recruit and activate immune cells, although the mechanism underlying the activity of YAP1-acitvated tubular cells in exaggerating the immune response and boosting DKD development remains to be elucidated yet. Interestingly, two small molecules with the highest binding affinities with YAP1 were identified using the virtual screening and molecular docking analysis, which underpins and warrants more research to unveil the potential of YAP1 as one potential therapeutic target for DKD.

Among these five hub genes, there is still little known about what roles and mechanisms *DYRK2*, *ATAD2* and *CHD3* play in the pathogenesis of DKD. DYRK2 is a serine/threonine kinase that regulates cell apoptosis in response to DNA damage through effectively phosphorylating p53 at Ser46 (Yogosawa et al., 2021). ATAD2 has genome-regulatory activities, like cell growth, differentiation and death (Wu et al., 2014), while CHD3 contributes to the chromatin remodeling by deacetylating histones that is required for a variety of activities like transcription (Sharapova et al., 2020). That’s to say, although our results suggested that these five novel hub genes have correlations with infiltrating immune cells and the development of DKD, further efforts and investigation in numerous areas are urgently needed if these newly discovered hub genes are highlighted as promising biomarkers or therapeutic targets for the monitoring and treatment of disease to succeed.

Our research has some significant limitations. Firstly, our study was constrained by the sample size, and more samples from different platforms could have increased the robustness of the findings. Secondly, experiments to evaluate the immune cells infiltration within renal microenvironment could have yielded a more persuasive conclusion, considering our bioinformatics analysis has also shown the dramatically elevated infiltrating immune cells. Finally, the close link between the five newly discovered hub genes with infiltrating NK cells by WGCNA and small-molecule inhibitors could have been validated by extensive experimental and clinical validation to dig deeper insight on the potential mechanism. Accordingly, future studies to verify the roles of these hub genes in orchestrating immune cell responses, as well as early diagnosis, risk stratification and monitoring of DKD patients, are urgently required before advancements can be made.

## Conclusion

In conclusion, we identified novel key genes and potential candidate small molecule drugs in DKD by integrated bioinformatics analysis. We for the first time identified *NFKB1*, *DYRK2*, *ATAD2, YAP1*, and *CHD3* as five newly discovered hub genes closely associated with the occurrence and development of DKD. Besides, significantly elevated YAP1 transcripts and protein levels were validated in cultured tubule cells and diabetic mice kidney, and two small-molecule drugs were identified as potential inhibitors for YAP1 to treat DKD. Extensive investigation of these hub genes revealed their capacity to trigger immune cells activation and distinguish DKD patients from healthy living donors, highlighting the potential of these hub genes as new biomarkers and therapeutic targets for the monitoring and treatment of DKD.

## Data Availability

The DKD-related microarray datasets (GSE131882, GSE142025, GSE30122, GSE96804, GSE104954) were retrieved from the GEO Datasets (https://www.ncbi.nlm.nih.gov/gds).

## Supplementary material

Supplementary Figure S1 The selection and filtration of cells based on QC metrics.

SSupplementary Figure S2 Quantification of infiltrating NK cells by MCP-counter technique showed robustly increased proportion of NK cells within kidney from both early stage DKD and advanced stage DKD samples.

Supplementary Figure S3 The determination of soft-thresholding power. **(A)** Histogram of connectivity distribution when beta=16. **(B)** Checking the scale free topology when beta = 16.

Supplementary Figure S4 The expression patterns of hub genes among normal control and DKD samples in GSE30122.

Supplementary Figure S5 The expression patterns of hub genes among normal control and DKD samples in GSE96804.

Supplementary Figure S6 The expression patterns of hub genes among normal control and DKD samples in GSE104954.

Supplementary Table S1 GO enrichment analysis of the commonly shared DEGs.

Supplementary Table S2 KEGG enrichment analysis of the commonly shared DEGs.

## References

[B1] Alicic R. Z. Rooney M. T. Tuttle K. R. (2017). Diabetic kidney disease: challenges, progress, and possibilities. Clin. J. Am. Soc. Nephrol. 12, 2032–2045. 10.2215/CJN.11491116 28522654 PMC5718284

[B2] Bash L. D. Selvin E. Steffes M. Coresh J. Astor B. C. (2008). Poor glycemic control in diabetes and the risk of incident chronic kidney disease even in the absence of albuminuria and retinopathy: atherosclerosis Risk in Communities (ARIC) Study. Arch. Intern. Med. 168, 2440–2447. 10.1001/archinte.168.22.2440 19064828 PMC2766035

[B3] Becht E. Giraldo N. A. Lacroix L. Buttard B. Elarouci N. Petitprez F. (2016). Estimating the population abundance of tissue-infiltrating immune and stromal cell populations using gene expression. Genome Biol. 17, 218. 10.1186/s13059-016-1070-5 27765066 PMC5073889

[B4] Bjorkstrom N. K. Ljunggren H. G. Michaelsson J. (2016). Emerging insights into natural killer cells in human peripheral tissues. Nat. Rev. Immunol. 16, 310–320. 10.1038/nri.2016.34 27121652

[B5] Bjornstad P. Cherney D. Maahs D. M. (2014). Early diabetic nephropathy in type 1 diabetes: new insights. Curr. Opin. Endocrinol. Diabetes Obes. 21, 279–286. 10.1097/MED.0000000000000074 24983394 PMC4138314

[B6] Bjornstad P. Cherney D. Z. Maahs D. M. (2015). Update on estimation of kidney function in diabetic kidney disease. Curr. Diab. Rep. 15, 57. 10.1007/s11892-015-0633-2 26188736 PMC6429956

[B7] Callaghan B. C. Little A. A. Feldman E. L. Hughes R. A. (2012). Enhanced glucose control for preventing and treating diabetic neuropathy. Cochrane Database Syst. Rev. 6, CD007543. 10.1002/14651858.CD007543 PMC4048127 22696371

[B8] Chen J J. Wang X. He Q. Bulus N. Fogo A. B. Zhang M. Z. (2020). YAP activation in renal proximal tubule cells drives diabetic renal interstitial fibrogenesis. Diabetes 69, 2446–2457. 10.2337/db20-0579 32843569 PMC7576565

[B9] Chen L. Wu B. Wang S. Xiong Y. Zhou B. Cheng X. (2020). Identification of cooperative gene regulation among transcription factors, LncRNAs, and MicroRNAs in diabetic nephropathy progression. Front. Genet. 11, 1008. 10.3389/fgene.2020.01008 33088282 PMC7490338

[B10] Dallakyan S. Olson A. J. (2015). Small-molecule library screening by docking with PyRx. Methods Mol. Biol. 1263, 243–250. 10.1007/978-1-4939-2269-7_19 25618350

[B11] de Melo T. R. de Souza K. S. C. Ururahy M. A. G. Bortolin R. H. Bezerra J. F. de Oliveira Galvao M. F. (2022). Toll-like receptor inflammatory cascade and the development of diabetic kidney disease in children and adolescents with type 1 diabetes. J. Paediatr. Child. Health 58, 996–1000. 10.1111/jpc.15884 35006634

[B12] Dounousi E. Duni A. Leivaditis K. Vaios V. Eleftheriadis T. Liakopoulos V. (2015). Improvements in the management of diabetic nephropathy. Rev. Diabet. Stud. 12, 119–133. 10.1900/RDS.2015.12.119 26676665 PMC5397987

[B13] Fan Y. Yi Z. D'Agati V. D. Sun Z. Zhong F. Zhang W. (2019). Comparison of kidney transcriptomic profiles of early and advanced diabetic nephropathy reveals potential new mechanisms for disease progression. Diabetes 68, 2301–2314. 10.2337/db19-0204 31578193 PMC6868471

[B14] Fineberg D. Jandeleit-Dahm K. A. Cooper M. E. (2013). Diabetic nephropathy: diagnosis and treatment. Nat. Rev. Endocrinol. 9, 713–723. 10.1038/nrendo.2013.184 24100266

[B15] Foresto-Neto O. Albino A. H. Arias S. C. A. Faustino V. D. Zambom F. F. F. Cenedeze M. A. (2020). NF-κB system is chronically activated and promotes glomerular injury in experimental type 1 diabetic kidney disease. Front. Physiol. 11, 84. 10.3389/fphys.2020.00084 32116790 PMC7026681

[B16] Grayson P. C. Eddy S. Taroni J. N. Lightfoot Y. L. Mariani L. Parikh H. (2018). Metabolic pathways and immunometabolism in rare kidney diseases. Ann. Rheum. Dis. 77, 1226–1233. 10.1136/annrheumdis-2017-212935 29724730 PMC6045442

[B17] Herzallah R. Karny M. (2011). Fully probabilistic control design in an adaptive critic framework. Neural Netw. 24, 1128–1135. 10.1016/j.neunet.2011.06.006 21752597

[B18] Jumper J. Evans R. Pritzel A. Green T. Figurnov M. Ronneberger O. (2021). Highly accurate protein structure prediction with AlphaFold. Nature 596, 583–589. 10.1038/s41586-021-03819-2 34265844 PMC8371605

[B19] Langfelder P. Horvath S. (2008). Wgcna: an R package for weighted correlation network analysis. BMC Bioinforma. 9, 559. 10.1186/1471-2105-9-559 PMC2631488 19114008

[B20] Li B. Li S. Fan Y. Diao H. Ye S. Peng H. (2022). Computational analysis reveals the characteristics of immune cells in glomerular and tubulointerstitial compartments in IgA nephropathy patients. Front. Genet. 13, 838863. 10.3389/fgene.2022.838863 35601494 PMC9116531

[B21] Li B. Tang Y. Ni X. Chen W. (2020). Immune cell landscape identification associates intrarenal mononuclear phagocytes with onset and remission of lupus nephritis in NZB/W mice. Front. Genet. 11, 577040. 10.3389/fgene.2020.577040 33304383 PMC7693546

[B22] Matoba K. Takeda Y. Nagai Y. Kawanami D. Utsunomiya K. Nishimura R. (2019). Unraveling the role of inflammation in the pathogenesis of diabetic kidney disease. Int. J. Mol. Sci. 20, E3393. 10.3390/ijms20143393 31295940 PMC6678414

[B23] Mensah-Brown E. P. Obineche E. N. Galadari S. Chandranath E. Shahin A. Ahmed I. (2005). Streptozotocin-induced diabetic nephropathy in rats: the role of inflammatory cytokines. Cytokine 31, 180–190. 10.1016/j.cyto.2005.04.006 15975818

[B24] Moon J. Y. Jeong K. H. Lee T. W. Ihm C. G. Lim S. J. Lee S. H. (2012). Aberrant recruitment and activation of T cells in diabetic nephropathy. Am. J. Nephrol. 35, 164–174. 10.1159/000334928 22286547

[B25] Na J. Sweetwyne M. T. Park A. S. Susztak K. Cagan R. L. (2015). Diet-induced podocyte dysfunction in drosophila and mammals. Cell Rep. 12, 636–647. 10.1016/j.celrep.2015.06.056 26190114 PMC4532696

[B26] Nathan D. M. Bayless M. Cleary P. Genuth S. Gubitosi-Klug R. Lachin J. M. (2013). Diabetes control and complications trial/epidemiology of diabetes interventions and complications study at 30 years: advances and contributions. Diabetes 62, 3976–3986. 10.2337/db13-1093 24264395 PMC3837056

[B27] Ogurtsova K. da Rocha Fernandes J. D. Huang Y. Linnenkamp U. Guariguata L. Cho N. H. (2017). IDF diabetes Atlas: global estimates for the prevalence of diabetes for 2015 and 2040. Diabetes Res. Clin. Pract. 128, 40–50. 10.1016/j.diabres.2017.03.024 28437734

[B28] Pan Y. Jiang S. Hou Q. Qiu D. Shi J. Wang L. (2018). Dissection of glomerular transcriptional profile in patients with diabetic nephropathy: SRGAP2a protects podocyte structure and function. Diabetes 67, 717–730. 10.2337/db17-0755 29242313

[B29] Robin X. Turck N. Hainard A. Tiberti N. Lisacek F. Sanchez J. C. (2011). pROC: an open-source package for R and S+ to analyze and compare ROC curves. BMC Bioinforma. 12, 77. 10.1186/1471-2105-12-77 PMC3068975 21414208

[B30] Robinson M. D. McCarthy D. J. Smyth G. K. (2010). edgeR: a bioconductor package for differential expression analysis of digital gene expression data. Bioinformatics 26, 139–140. 10.1093/bioinformatics/btp616 19910308 PMC2796818

[B31] Said S. M. Nasr S. H. (2016). Silent diabetic nephropathy. Kidney Int. 90, 24–26. 10.1016/j.kint.2016.02.042 27312444

[B32] Sharapova S. O. Golovataya E. I. Shepelevich E. V. Mareika Y. E. Guryanova I. E. Stegantseva M. V. (2020). Nijmegen breakage syndrome in two half sibs with peripheral T-cell lymphoma and cortical T-cell acute lymphoid leukemia. Cent. Eur. J. Immunol. 45, 507–510. 10.5114/ceji.2020.103387 33658897 PMC7882413

[B33] Song Z. Gao P. Zhong X. Li M. Wang M. Song X. (2022). Identification of five hub genes based on single-cell RNA sequencing data and network pharmacology in patients with acute myocardial infarction. Front. Public Health 10, 894129. 10.3389/fpubh.2022.894129 35757636 PMC9219909

[B34] Sterling T. Irwin J. J. (2015). ZINC 15--ligand discovery for everyone. J. Chem. Inf. Model. 55, 2324–2337. 10.1021/acs.jcim.5b00559 26479676 PMC4658288

[B35] Tang S. C. W. Yiu W. H. (2020). Innate immunity in diabetic kidney disease. Nat. Rev. Nephrol. 16, 206–222. 10.1038/s41581-019-0234-4 31942046

[B36] Turner J. E. Rickassel C. Healy H. Kassianos A. J. (2019). Natural killer cells in kidney health and disease. Front. Immunol. 10, 587. 10.3389/fimmu.2019.00587 30972076 PMC6443628

[B37] Uchida T. Ito S. Kumagai H. Oda T. Nakashima H. Seki S. (2019). Roles of natural killer T cells and natural killer cells in kidney injury. Int. J. Mol. Sci. 20, E2487. 10.3390/ijms20102487 31137499 PMC6567827

[B38] Winter L. Wong L. A. Jerums G. Seah J. M. Clarke M. Tan S. M. (2018). Use of readily accessible inflammatory markers to predict diabetic kidney disease. Front. Endocrinol. 9, 225. 10.3389/fendo.2018.00225 PMC5992400 29910771

[B39] Wu G. Lu X. Wang Y. He H. Meng X. Xia S. (2014). Epigenetic high regulation of ATAD2 regulates the Hh pathway in human hepatocellular carcinoma. Int. J. Oncol. 45, 351–361. 10.3892/ijo.2014.2416 24805933

[B40] Yogosawa S. Ohkido M. Horii T. Okazaki Y. Nakayama J. Yoshida S. (2021). Mice lacking DYRK2 exhibit congenital malformations with lung hypoplasia and altered Foxf1 expression gradient. Commun. Biol. 4, 1204. 10.1038/s42003-021-02734-6 34671097 PMC8528819

[B41] Yung S. Chau M. K. Zhang Q. Zhang C. Z. Chan T. M. (2013). Sulodexide decreases albuminuria and regulates matrix protein accumulation in C57BL/6 mice with streptozotocin-induced type I diabetic nephropathy. PloS one 8, e54501. 10.1371/journal.pone.0054501 23349910 PMC3551764

[B42] Zheng Z. Li C. Shao G. Li J. Xu K. Zhao Z. (2021). Hippo-YAP/MCP-1 mediated tubular maladaptive repair promote inflammation in renal failed recovery after ischemic AKI. Cell Death Dis. 12, 754. 10.1038/s41419-021-04041-8 34330891 PMC8324794

