# Supplementary figures and images for: Identification of novel key genes and potential candidate small molecule drugs in diabetic kidney disease using comprehensive bioinformatics analysis
Source: Front Genet. 2022 Aug 12;13:934555. doi: 10.3389/fgene.2022.934555 (PMC9411649; doi:10.3389/fgene.2022.934555)

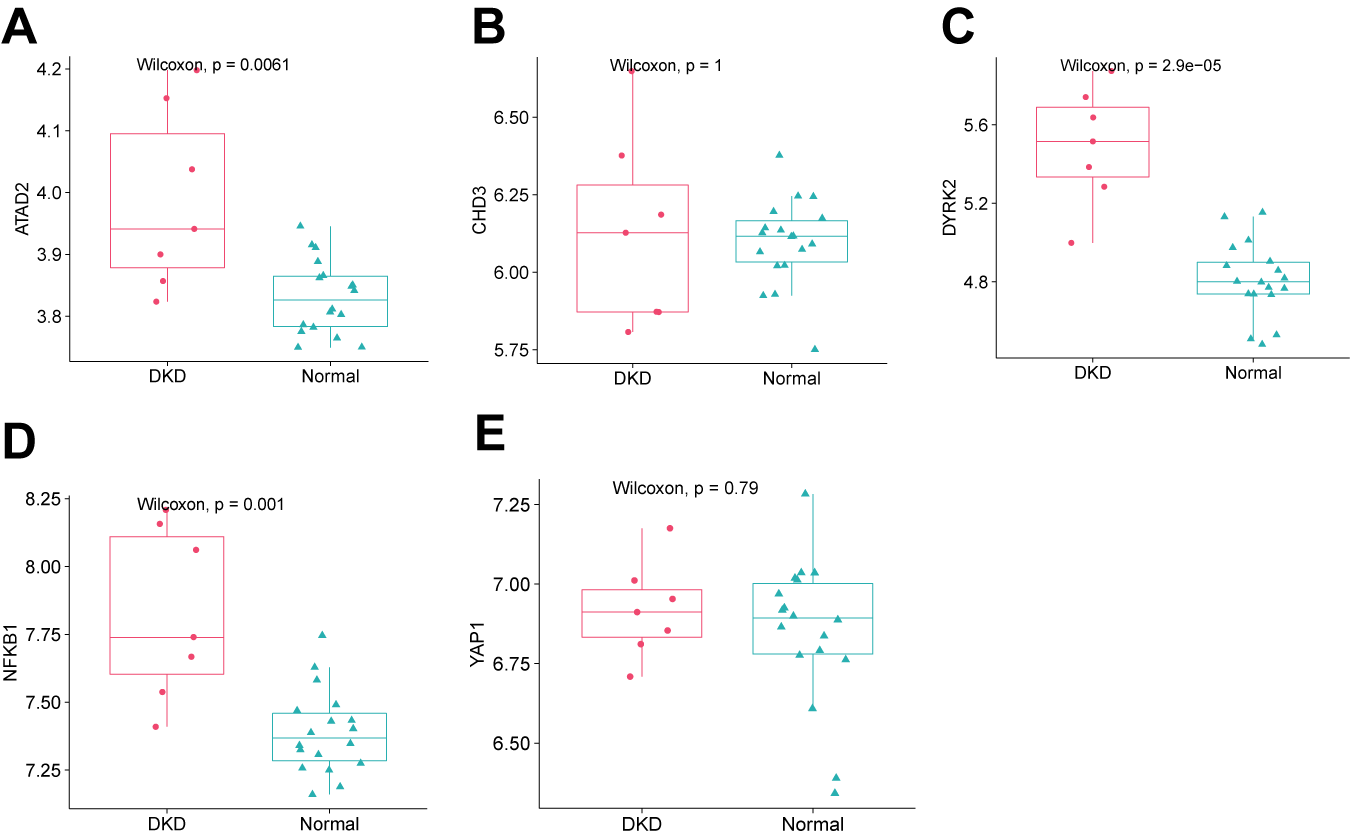

Supplement: Supplementary file 1 [file Image6.TIF]

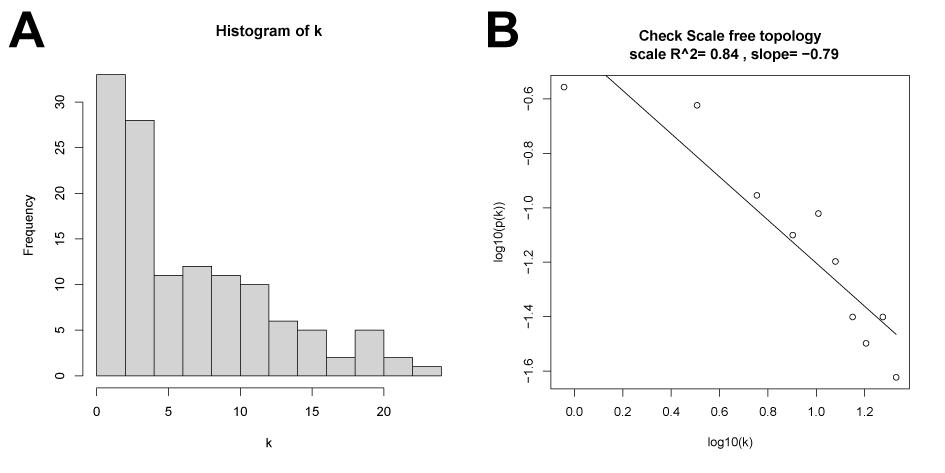

Supplement: Supplementary file 3 [file Image3.TIF]

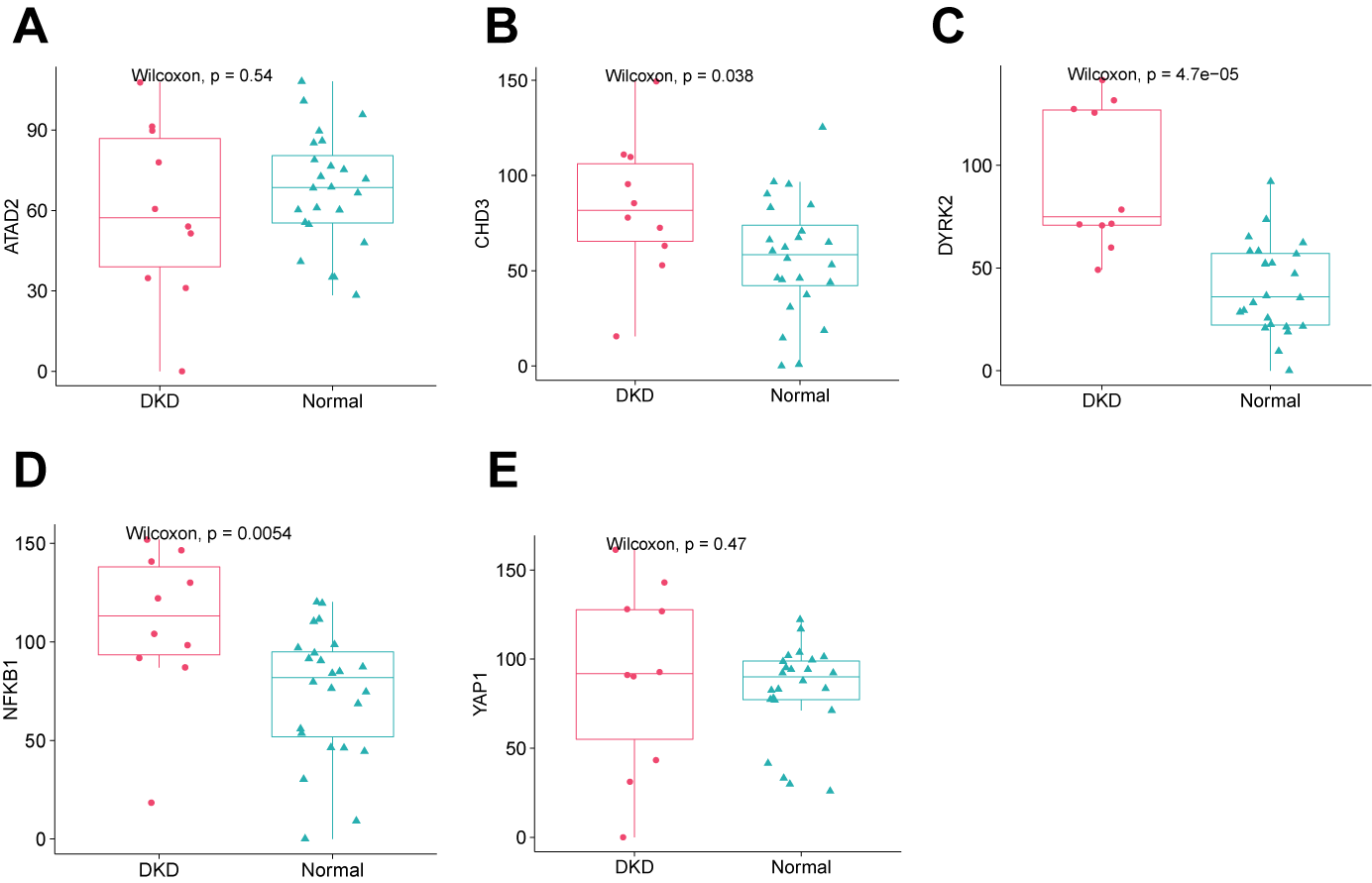

Supplement: Supplementary file 4 [file Image4.TIF]

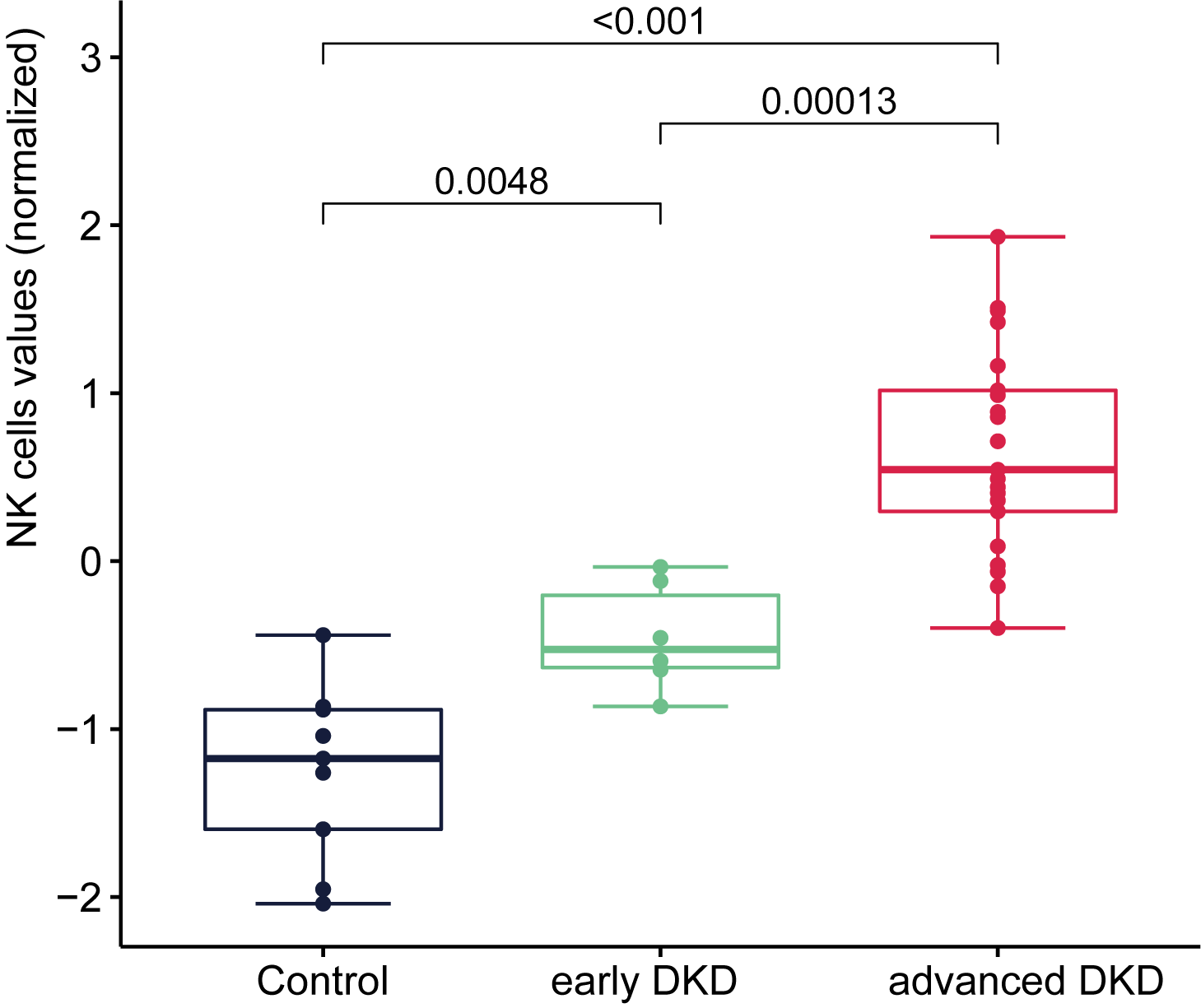

Supplement: Supplementary file 5 [file Image2.TIF]

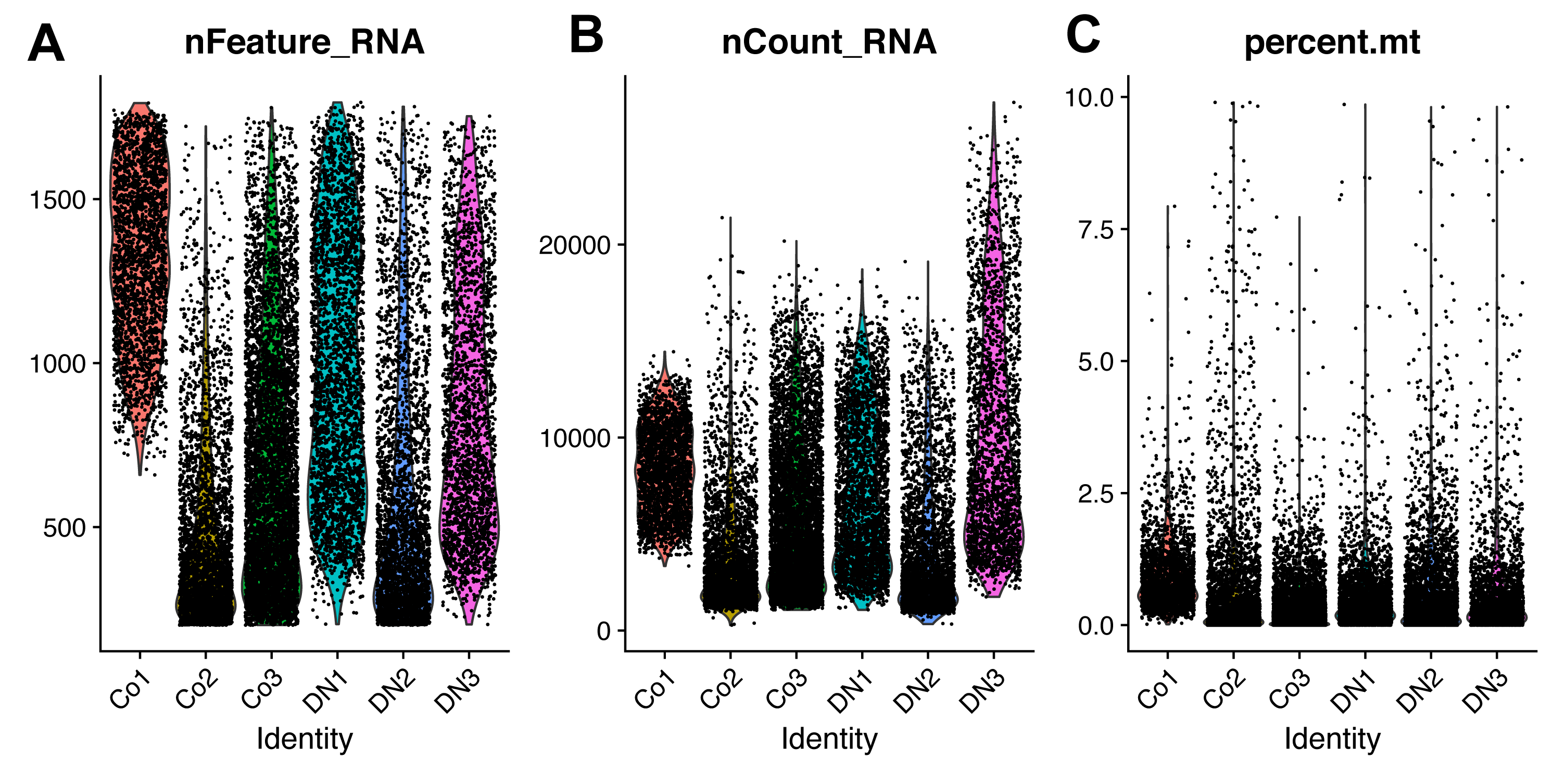

Supplement: Supplementary file 6 [file Image1.TIF]

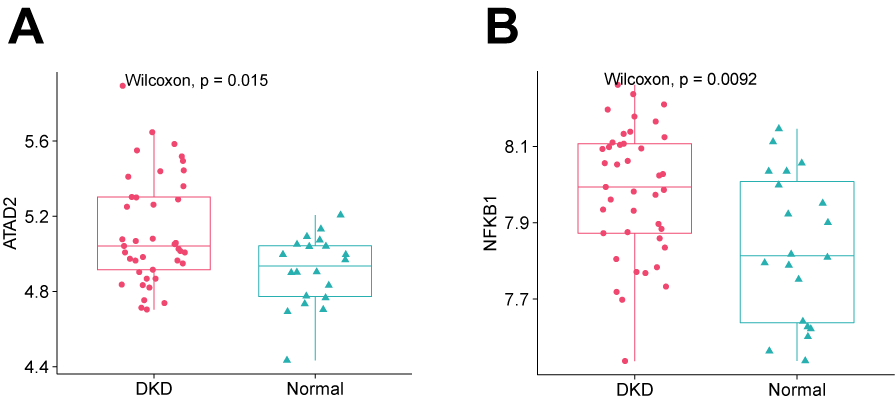

Supplement: Supplementary file 8 [file Image5.TIF]
