# Supplementary material for: Identification of novel key genes and potential candidate small molecule drugs in diabetic kidney disease using comprehensive bioinformatics analysis
Source: Front Genet. 2022 Aug 12;13:934555. doi: 10.3389/fgene.2022.934555 (PMC9411649; doi:10.3389/fgene.2022.934555)
Supplement: Supplementary file 2 [file Table1.DOCX]

**Supplementary Table 1. GO enrichment analysis of the commonly shared DEGs.**

| **ID** | **Description** | **p-value** | **Count** |
| --- | --- | --- | --- |
| GO:0043312 | neutrophil degranulation | <0.001 | 17 |
| GO:0002283 | neutrophil activation involved in immune response | <0.001 | 17 |
| GO:0002446 | neutrophil mediated immunity | <0.001 | 17 |
| GO:0042119 | neutrophil activation | <0.001 | 17 |
| GO:0038093 | Fc receptor signaling pathway | <0.001 | 12 |
| GO:0002429 | immune response-activating cell surface receptor signaling pathway | <0.001 | 16 |
| GO:0002757 | immune response-activating signal transduction | <0.001 | 16 |
| GO:0042110 | T cell activation | <0.001 | 15 |
| GO:0006909 | phagocytosis | <0.001 | 13 |
| GO:0001706 | endoderm formation | <0.001 | 6 |
| GO:1903131 | mononuclear cell differentiation | <0.001 | 13 |
| GO:1903039 | positive regulation of leukocyte cell-cell adhesion | <0.001 | 10 |
| GO:0034113 | heterotypic cell-cell adhesion | <0.001 | 6 |
| GO:0007159 | leukocyte cell-cell adhesion | <0.001 | 12 |
| GO:0032496 | response to lipopolysaccharide | <0.001 | 11 |
