# Supplementary material for: Identification of novel key genes and potential candidate small molecule drugs in diabetic kidney disease using comprehensive bioinformatics analysis
Source: Front Genet. 2022 Aug 12;13:934555. doi: 10.3389/fgene.2022.934555 (PMC9411649; doi:10.3389/fgene.2022.934555)
Supplement: Supplementary file 7 [file Table2.DOCX]

**Supplementary Table 2. KEGG enrichment analysis for the commonly shared DEGs.**

| **ID** | **Description** | **p-value** | **Count** |
| --- | --- | --- | --- |
| hsa05140 | Leishmaniasis | <0.001 | 7 |
| hsa05135 | Yersinia infection | <0.001 | 8 |
| hsa04666 | Fc gamma R-mediated phagocytosis | <0.001 | 7 |
| hsa04662 | B cell receptor signaling pathway | <0.001 | 6 |
| hsa04380 | Osteoclast differentiation | <0.001 | 7 |
| hsa05417 | Lipid and atherosclerosis | <0.001 | 8 |
| hsa04668 | TNF signaling pathway | <0.001 | 6 |
| hsa05133 | Pertussis | <0.001 | 5 |
| hsa05152 | Tuberculosis | <0.001 | 7 |
| hsa04514 | Cell adhesion molecules | <0.001 | 6 |
| hsa01522 | Endocrine resistance | <0.001 | 5 |
| hsa04810 | Regulation of actin cytoskeleton | <0.001 | 7 |
| hsa04660 | T cell receptor signaling pathway | <0.001 | 5 |
| hsa05166 | Human T-cell leukemia virus 1 infection | <0.001 | 7 |
| hsa04670 | Leukocyte transendothelial migration | <0.001 | 5 |
